# Preoperative sonographic axillary staging in breast cancer: correlation with sentinel node sampling

**DOI:** 10.1186/bcr2985

**Published:** 2011-11-04

**Authors:** P Sankaye, S Chhatani, S Doyle, J Steel, K Paisley, B Lyons, R Watkins, G Porter

**Affiliations:** 1 Plymouth Hospitals NHS Trust, Plymouth, UK

## Introduction

Axillary ultrasound staging with core biopsy (CB) or fine needle aspiration (FNA) in primary breast cancer is well established. Negative patients will have a sentinel lymph node biopsy (SLNB). This study compares the initial ultrasound finding versus final axillary histology in patients undergoing SLNB.

## Methods

A total of 249 breast carcinoma patients, who underwent SLNB between August 2007 and January 2011, were included. Axillary ultrasound and histology results were reviewed. Ultrasound findings and any subsequent biopsies were recorded in positive and negative SLNB groups. The axillary lymph node biopsy histology slides were reviewed in the false negative axillary ultrasound biopsy/FNA group.

## Results

**Figure 1:**
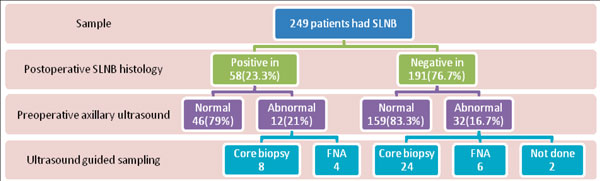


Of 249 patients, 191 (76.7%) were SLNB-negative and 58 (23.3%) were SLNB-positive. Thirty out of 191 (16%) patients without axillary metastases had ultrasound-guided sampling. Two out of 191 did not have ultrasound-guided sampling as the procedure was deemed unsafe. Twelve out of 58 (21%) SLNB-positive patients had abnormal ultrasound appearances; 8/12(67%) had CB and 4/12 (33%) had FNA, not significantly different to the CB/FNA rates in SLNB-negative patients (24/32 (75%) and 6/32 (25%), respectively; *P *= 0.2). See Figure 1. Review of 12 false negative histology slides revealed 1/12 (8%) had micrometastasis and 11/12 (92%) were benign.

## Conclusion

There are no significant differences in the rate of axillary ultrasound abnormality between SLNB-positive and SLNB-negative patients. This suggests that ultrasound nodal abnormalities due to malignancy are probably being diagnosed by ultrasound-guided sampling and do not progress to SLNB. It also supports SLNB for sonographically abnormal lymph nodes as opposed to axillary nodal dissection, as many of these patients will not have metastases, if USS sampling is negative. Our small study has not shown significant benefit of CB over FNA in axillary staging. We found a low upgrade rate on reviewing original ultrasound-guided histology slides, supporting current pathology techniques.

